# Inpatient Burden of Prurigo Nodularis in the United States

**DOI:** 10.3390/medicines6030088

**Published:** 2019-08-11

**Authors:** Katherine A. Whang, Sewon Kang, Shawn G. Kwatra

**Affiliations:** 1 Department of Dermatology, Johns Hopkins University School of Medicine, Baltimore, MD 21205, USA; 2 Department Bloomberg School of Public Health, Johns Hopkins Bloomberg School of Public Health, Baltimore, MD 21205, USA

**Keywords:** prurigo nodularis, pruritus, itch, inpatient, disease burden, national inpatient sample

## Abstract

**Background:** Although prurigo nodularis (PN) has a significant burden of disease, little is known about its epidemiology and disease burden within the United States. We describe the characteristics of hospitalized patients diagnosed with PN and assess the factors associated with hospitalization. **Methods:** We performed a cross-sectional study of the 2016 National Inpatient Sample, a representative sample of 20% of hospital discharges nationally. **Results:** Patients diagnosed with PN accounted for 3.7 inpatient visits per 100,000 discharges nationally in 2016. Patients with PN were more likely to be black (odds ratio (OR) 4.43, 95% CI (3.33–6.08), *p* < 0.001) or Asian (OR 3.44, 95% CI (1.39–5.08), *p* = 0.003) compared with white patients. Patients diagnosed with PN had both a longer length of hospital stay (mean ± SD, 6.51 ± 0.37 days vs. 4.62 ± 0.02 days, *p* < 0.001) and higher cost of care ($14,772 ± $964 vs. $11,728 ± $106, *p* < 0.001) compared with patients without PN. Patients with PN were significantly more likely to be admitted for HIV complications (OR 78.2, 95% CI (46.4–131.8), *p* < 0.001). PN contributes to increased inpatient cost of care and length of hospitalization. **Conclusions:** There are racial disparities associated with hospital admission of patients diagnosed with PN.

## 1. Introduction

Prurigo nodularis (PN) is a chronic pruritic condition that is characterized by repeated scratching behavior and the presence of multiple, intensely itchy nodules [1]. These lesions often are excoriated to the point of ulceration and are symmetrically distributed on the extensor surfaces of the limbs and trunk [2]. PN has a significant impact on the quality of life and is linked with numerous systemic and psychological comorbidities, such as anxiety, depression, and sleep disturbance, as has been found with other pruritic conditions, like atopic dermatitis [3,4,5,6,7]. Recent studies on the pathogenetic mechanisms of PN has revealed the importance of pro-inflammatory cytokines, such as IL-31, and neuropeptides in lesional skin that may contribute to altered nerve density and increased inflammation in PN [8,9,10]. Despite the tremendous burden of disease of PN, there is very little known about the etiology and epidemiology of PN. A German study examined 108 PN patients in a predominantly Caucasian population and observed a female predominance (64%) with a median age of 61.5 years and found that more than half of PN cases had an atopic predisposition or concomitant atopic dermatitis [2]. In fact, Tanaka et al describe two distinct forms of PN: An early-onset atopic form and a late-onset non-atopic form [11]. Our group also recently described a cohort of PN patients seen in the Johns Hopkins Health System, though epidemiologic conclusions are difficult to draw based on a single health system experience [5,12]. 

One potential reason for the limited amount of epidemiologic data available is that PN is fairly uncommon, and in the past was grouped together with other chronic pruritic conditions in previous disease classification systems [1]. However, in 2015, the United States transitioned to the new disease classification system, International Classification of Diseases, Tenth Revision, Clinical Modification (ICD-10-CM), from the previously used ICD-9-CM, allowing for increased specificity in disease coding. For the first time, PN received a dedicated diagnosis code, enabling new avenues for epidemiologic research on PN to be explored. 

Previous studies have demonstrated an association between PN and several comorbidities, such as HIV infection, hepatitis, congestive heart failure, chronic kidney disease, Type 2 diabetes mellitus, and several psychiatric conditions [5]. These conditions contribute to PN’s burden of disease and may affect hospitalization and cost of care. Due to the limited research on PN as a disease entity, little is known on the inpatient burden of PN in the United States. In the current study, we analyze the epidemiology and inpatient burden of PN in the United States, as well as factors associated with hospitalization for PN in a nationally representative database of hospitalizations in 2016.

## 2. Materials and Methods

### 2.1. Data Source

We analyzed data from the National Inpatient Sample (NIS) from 2016. The NIS is administered by the Healthcare Cost and Utilization Project (HCUP) of the Agency for Healthcare Research and Quality. Each year of data represented a stratified sample of approximately 20% of all inpatient hospitalizations within the United States. Of note, 2016 was the first complete year that prurigo nodularis received an independent diagnostic code as detailed below. Sample weights provided by NIS were used to account for sampling design of hospitals to determine nationally representative estimates. The study was exempt from the institutional review board because the database was deidentified before use.

### 2.2. Selection of Prurigo Nodularis Cohort

Patients with a diagnosis of prurigo nodularis were selected using ICD-10-CM codes. An ICD-10-CM code of L28.1 corresponded to a diagnosis of prurigo nodularis. 

### 2.3. Statistical Analysis 

Data analysis was performed using survey models that accounted for NIS-provided survey weights, sampling clusters, and strata using Stata version 15 (StataCorp, College Station, TX, USA). We determined the estimated prevalence of hospitalization for PN. Cost of inpatient care was determined based on the total reported charge of hospitalization and the all-payer inpatient cost-to-charge ratio estimates for hospitals provided by HCUP. 

The control group included all discharges with no diagnosis of PN, representing the population of hospitalized patients in the United States. In order to determine risk factors for hospitalization of patients diagnosed with PN, binary logistic models were constructed using hospitalization with PN as the dependent variable. Independent variables included age, gender, race/ethnicity, the median annual income of the hospital ZIP code, health insurance type, season of admission, hospital location, teaching status, and hospital bed capacity. A *p*-value of <0.05 was considered significant with Bonferroni correction.

## 3. Results

There was a total of 7,135,090 discharges reported in the NIS dataset during 2016. There were 265 hospitalized patients with a diagnosis of PN (weighted 1325, 95% confidence interval (CI) (1120–1530)) or 3.7 inpatient visits per 100,000 discharges in 2016.

### 3.1. Factors Associated with Hospitalization for Prurigo Nodularis 

**Figure 1:**
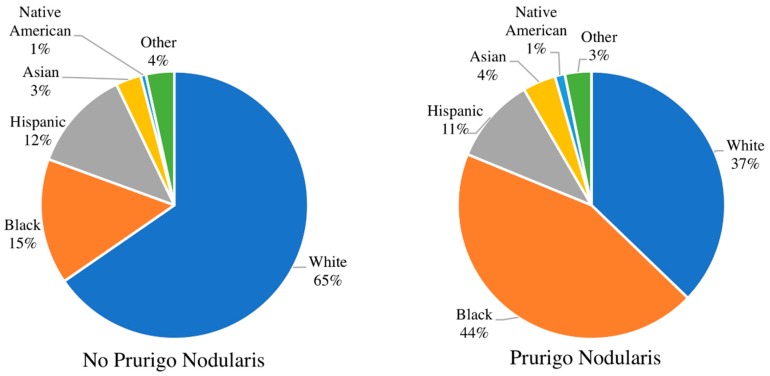
Comparison of racial distribution. The racial distribution of patients with no prurigo nodularis (**left**) compared to patients with prurigo nodularis (**right**). The population of prurigo nodularis patients is disproportionately black compared to patients with no prurigo nodularis.

Patients admitted as inpatients to hospitals with a diagnosis of PN were on average older than patients without a diagnosis of PN (mean ± SD 55.2 ± 0.9 years vs. 49.0 ± 0.2 years, respectively). Patients with PN were more likely to be black (survey-weighted logistic regression; odds ratio (OR) 4.43, 95% CI (3.33–6.08), *p* < 0.001), Hispanic (OR 1.77, 95% CI (1.09–2.88), *p* = 0.02), or Asian (OR 3.44, 95% CI (1.39–5.08), *p* = 0.003) compared with white patients (Figure 1). Black PN patients were more likely to be male than black patients of the general patient population (59.1% vs. 41.1%; OR 1.97, 95% CI (1.36–2.85), *p* = 0.0003). PN patients were also more likely to have Medicare (OR 2.81, 95% CI (1.80–4.39), *p* < 0.001) or Medicaid (OR 2.24, 95% CI (1.44–3.47), *p* < 0.001), as compared to patients with private insurance. In the US, Medicare and Medicaid are government-run programs that provide health insurance to the elderly and low-income. Hospitalized patients with PN were more likely to be seen in teaching hospitals (OR 2.60, 95% CI (1.76–3.84), *p* < 0.001) compared to non-teaching and hospitals with a large bed capacity (OR 2.15, 95% CI (1.41–3.27), *p* < 0.001). 

In multivariate logistic regression models with stepwise elimination, age, race/ethnicity, insurance status, teaching status, and hospital bed size were found to have a statistically significant contribution to hospitalization for PN (Table 1). The factors of sex, income quartile, season, and hospital region were found to have no statistically significant contribution to hospitalization for PN.

### 3.2. Length of Stay and Cost of Care

The length of stay (LOS) in the hospital was longer in duration for patients with a diagnosis of PN compared with patients without PN (mean ± SD, 6.51 ± 0.37 days vs. 4.62 ± 0.02 days, *p* < 0.001). In multivariate linear regression for LOS in patients diagnosed with PN, older age (≥18 years) was associated with a longer duration of hospital stay (beta coefficient = 3.46, 95% CI [0.93–5.99], *p* = 0.007) (Table 2). 

The total cost of care for hospitalized patients diagnosed with PN was $18,686,522 in 2016; however, the actual total cost was higher because 12 patients had missing values for cost. The average cost of care was higher in patients diagnosed with PN compared with patients without PN ($14,772 ± $964 vs. $11,728 ± $106, *p* < 0.001). In multivariate linear regression for cost of care in patients diagnosed with PN, older age (≥18 years) was associated with increased cost (age under 18 years: Beta coefficient = $8,980, 95% CI (2,271.54–15,688.7), *p* = 0.009) (Table 2).

### 3.3. Primary Reason for Admission of Prurigo Nodularis Patients

**Figure 2:**
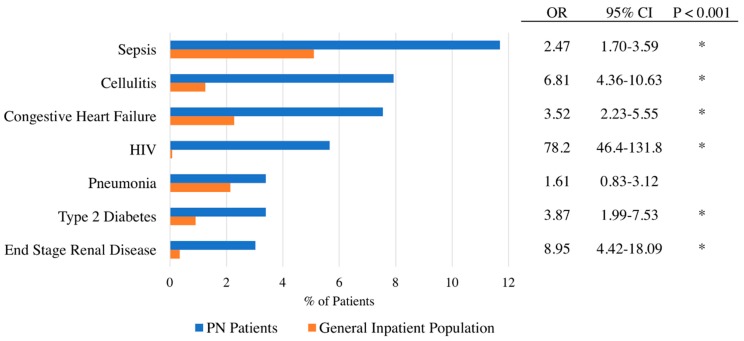
Comparison of primary admission reason. Primary admission reason for prurigo nodularis patients compared to patients with no prurigo nodularis.

The most common reasons for admission to the hospital for patients diagnosed with PN were sepsis (11.7%), cellulitis (7.9%), acute exacerbation of congestive heart failure (7.5%), HIV (5.7%), pneumonia (3.4%), and end stage renal disease (3.0%) (Figure 2). Patients with PN were significantly more likely to be admitted due to HIV complications compared to the general inpatient population (OR 78.2, 95% CI (46.4–131.8), *p* < 0.001). Furthermore, PN patients with concomitant HIV were significantly more likely to be black than white (OR 8.2, 95% CI (1.02–66.0), *p* = 0.048).

## 4. Discussion

This study represents the first epidemiological investigation of PN employing a national dataset in the United States to be reported in the literature. We estimate that approximately 1325 patients diagnosed with PN were hospitalized in the United States in 2016 or 3.7 inpatients per 100,000 discharges. These PN patients had both longer hospital stays and increased costs of care as compared to the general inpatient population. This is likely to be an underestimation of the overall PN inpatient burden as there is a lack of disease awareness among most physicians, and many patients do not receive inpatient dermatology consultations. We demonstrate that PN patients are more likely than the general population to be older in age, particularly in the 40 to 59 years age range and that increased age was associated with longer hospital stay and increased the cost of care. We attribute these age-related differences to the increased concomitant systemic comorbidities with age, such as diabetes mellitus and chronic kidney disease, which are systemic causes themselves of chronic pruritus and have been found to be associated with PN [5]. 

Our study also demonstrates increased rates of hospitalization in PN patients who were non-white, demonstrating racial disparities in the presentation of PN. In particular, these results confirmed recent findings indicating that PN disproportionately affected blacks [5]. Indeed, among the different races studied, blacks had the greatest likelihood of having PN. As previously described, black PN patients were also more likely than whites, Asians, or Hispanics to have concomitant HIV infection [5]. We found that black patients with a PN diagnosis were significantly more likely to be male compared to the general population of black patients. Recent findings have demonstrated that out of all new HIV diagnoses to black individuals in 2017, 76% were male, which may explain the increased likelihood of black males with PN [13]. 

Interestingly, infections including cellulitis and sepsis were frequent reasons for admission among patients with PN. Excoriation of skin nodules to the point of ulceration may serve as a nidus for infection in PN patients. In addition, given that PN is reported to have a positive predictive value for a CD4 count of <200 among HIV patients [14], PN patients are also likely to be at greater risk of infection because of more frequent rates of immunosuppression. Increased frequency of end stage renal disease and congestive heart failure as reasons for admission are also consistent with prior studies showing an association between cardiovascular and renal comorbidities in patients with PN [5,15,16].

Strengths of this study include the use of a nationally representative dataset with over 7 million records. Given the recent transition from ICD-9-CM to ICD-10-CM, this is the first study that provides epidemiologic data on PN in the United States. Weaknesses include the small PN sample size given the under-recognition of PN by non-dermatologic specialties. Effects of disease severity and treatment on hospitalization could also not be determined. 

In conclusion, our study characterizes the inpatient burden of PN in the United States and demonstrates increased cost and length of hospital stay in the care of these patients. Our study also confirms racial differences in the burden of PN, with PN patients most likely to be black. PN patients were also found to be more likely to be admitted for infections and comorbid cardiovascular and renal conditions. Additional studies are needed to further characterize the ambulatory disease burden of PN.

## Figures and Tables

**Table 1:** Demographics of hospitalized patients with a diagnosis of prurigo nodularis compared to the general inpatient population without a diagnosis of prurigo nodularis.

Variable	General Inpatient Population without a Diagnosis of Prurigo Nodularis		Patients with Prurigo Nodularis		Adjusted OR	*p*-Value
	Est. Frequency	Percent (95% Confidence Interval)	Est. Frequency	Percent (95% Confidence Interval)		
**AGE, y**						
0–17	5,479,694	15.3 [14.9–15.9]	5	0.38 [0.05–2.64]	0.04	0.002
18–39	7,423,759	20.8 [20.5–21.0]	210	15.8 [11.8–20.9]	1.00	---
40–59	7,403,610	20.7 [20.5–21.0]	620	46.8 [40.2–53.5]	2.95	<0.001
60–79	10,515,272	29.4 [29.0–29.7]	390	29.4 [24.0–35.5]	1.34	0.263
>80	4,886,856	13.7 [13.5–13.9]	100	7.55 [4.83–11.60]	0.75	0.386
**RACE**						
White	22,141,260	65.4 [64.4–66.3]	464	37.2 [30.8–44.1]	1.00	---
Black	5,145,981	15.2 [14.6–15.8]	550	44.0 [37.1–51.1]	4.43	<0.001
Hispanic	4,150,991	12.3 [11.6–12.9]	130	10.4 [6.9–15.4]	1.77	0.02
Asian	1,037,020	3.06 [2.83–3.32]	50	4.00 [2.22–7.09]	2.66	0.003
Native American	219,690	0.65 [0.56–0.75]	15	1.20 [0.39–3.67]	3.44	0.036
Other	1,156,259	3.42 [3.12–3.74]	40	3.20 [1.63–6.20]	1.29	0.586
**GENDER**						
Female	15,439,345	43.3 [43.1–43.5]	635	47.9 [41.7–54.2]	1.00	---
Male	20,236,076	56.7 [56.5–56.9]	690	52.1 [45.8–58.3]	1.00	0.75
**SEASON**						
Winter	8,914,808	25.0 [25.0–25.0]	400	30.2 [24.9–36.1]	1.00	---
Spring	9,015,068	25.3 [25.2–25.3]	315	23.8 [19.3–29.0]	0.81	0.213
Summer	8,914,118	25.0 [25.0–25.0]	305	23.0 [18.2–28.7]	0.73	0.106
Fall	8,796,723	24.7 [24.6–24.7]	270	20.4 [15.9–25.8]	0.72	0.063
**INCOME QUARTILE**						
First	10,774,519	30.7 [29.8–31.6]	465	36.9 [30.4–43.9]	1.00	---
Second	8,915,683	25.4 [24.8–26.0]	270	21.4 [16.5–27.3]	0.98	0.929
Third	8,387,702	23.9 [23.4–24.5]	290	23.0 [17.9–29.1]	1.18	0.398
Fourth	6,999,502	20.0 [19.0–20.9]	235	18.7 [13.2–24.0]	1.38	0.112
**INSURANCE**						
Medicare	14,127,590	39.6 [39.1–40.1]	645	49.0 [42.8–55.4]	2.81	<0.001
Medicaid	8,241,094	23.1 [22.6–23.7]	370	28.1 [22.7–34.3]	2.24	<0.001
Private	10,734,828	30.1 [29.6–30.6]	205	15.6 [11.6–20.7]	1.00	---
Self-pay	1,377,983	3.87 [3.71–4.03]	65	4.94 [2.84–8.48]	1.61	0.238
No charge	111,960	0.31 [0.27–0.37]	0			---
Other	1,058,214	2.97 [2.77–3.19]	30	2.28 [1.03–4.96]	1.28	0.612
**INSURED**						
Yes	33,078,581	92.7 [92.5–92.9]	1,220	92.1 [88.0–94.9]	1.00	---
No	1,377,983	3.86 [3.70–4.03]	65	4.91 [2.81–8.42]	1.00	---
Other or no charge	1,170,174	3.28 [3.07–3.50]	30	2.26 [1.03–4.92]	1.00	---
**REGION**						
Northeast	6,599,084	18.5 [17.9–19.2]	270	20.4 [15.3–26.6]	1.00	---
Midwest	7,933,647	22.2 [21.6–22.9]	355	26.8 [20.2–34.6]	1.38	0.14
South	14,041,126	39.3 [38.5–40.1]	445	33.6 [26.6–41.3]	0.91	0.649
West	7,122,759	20.0 [19.4–20.6]	255	19.2 [14.0–25.9]	1.25	0.328
**TEACHING STATUS**						
Nonteaching	23,291,422	65.4 [64.7–66.1]	1,130	85.3 [79.9–89.4]	1.00	---
Teaching	12,320,949	34.6 [33.9–35.3]	195	14.7 [10.6–20.1]	2.60	<0.001
**HOSPITAL BED CAPACITY**						
Small	6,674,756	18.7 [18.1–19.3]	170	12.8 [9.1–17.7]	1.00	---
Medium	10,351,226	29.0 [28.4–29.7]	240	18.1 [13.3–24.2]	1.13	0.629
Large	18,632,207	52.2 [51.5–53.0]	915	69.1 [62.1–75.2]	2.15	<0.001

**Table 2:** Multivariate linear regression for the cost of care and length of stay among prurigo nodularis patients.

Demographic	Cost of Care			Length of Stay		
	Adjusted Beta	95% CI	*p*-Value	Adjusted Beta	95% CI	*p*-Value
**AGE, y**						
0–17	Reference					
18–39	8980.12	[2271.54–15688.7]	0.009	3.46	[0.93–5.99]	0.007
40–59	10306.25	[3295.591–17316.91]	0.004	2.72	[0.20–5.24]	0.034
60–79	17129.11	[8512.382–25745.83]	<0.001	3.74	[0.99–6.50]	0.008
>80	7701.64	[−1590.12–16993.42]	0.104	1.06	[−2.43–4.57]	0.55
**SEASON**						
Winter	Reference					
Spring	2428.98	[−3651.91–8509.88]	0.433	0.34	[−2.05–2.74]	0.776
Summer	−2276.29	[−6883.1–2330.51]	0.333	−2.08	[−4.08–−0.08]	0.041
Fall	−1365.68	[−6400.697–3669.33]	0.595	−2.06	[−4.28–0.15]	0.069
**GENDER**						
Female	Reference					
Male	−2927.89	[-7155.95–1300.16]	0.175	−0.23	[−1.84–1.38]	0.781
**RACE**						
White	Reference					
Black	−696.01	[−5725.16–4333.13]	0.786	0.18	[−2.06–2.42]	0.875
Hispanic	2806.11	[−4865.536–10477.76]	0.473	−0.28	[−2.97–2.42]	0.841
Asian	5474.29	[−1684.91–12633.5]	0.134	−0.38	[−3.27–2.50]	0.794
Native American	5100.59	[−9957.69–20158.88]	0.507	4.44	[−3.10–11.99]	0.248
Other	−6695.23	[−14271.62–881.14]	0.083	−2.55	[−5.51–0.41]	0.092
**INCOME QUARTILE**						
First	Reference					
Second	1357.71	[−4561.79–7277.22]	0.653	1.47	[−1.26–4.20]	0.29
Third	−326.29	[−4849.39–4196.79]	0.888	0.25	[−1.59–2.09]	0.793
Fourth	2211.26	[−5600.19–10022.73]	0.579	−0.41	[−2.73–1.90]	0.725
**INSURANCE**						
Medicare	3799.07	[−2029.32–9627.46]	0.201	2.14	[−0.31–4.59]	0.87
Medicaid	4274.12	[−666.47–9214.72]	0.09	2.14	[−0.07–4.35]	0.058
Private	Reference					
Self-pay	−200.49	[−6367.36–5966.37]	0.949	0.23	[−2.88–3.32]	0.886
No charge						
Other	355.35	[−8232.30–8943.01]	0.935	3.37	[−0.96–7.70]	0.127
**REGION**						
Northeast	Reference					
Midwest	1947.42	[−5246.55–9141.39]	0.596	−0.84	[−3.76–2.08]	0.573
South	951.9	[−4984.18–6888.00]	0.753	0.12	[−2.44–2.68]	0.929
West	2914.19	[−4681.94–10510.34]	0.452	−0.48	[−3.35–2.40]	0.745
**TEACHING STATUS**						
Nonteaching	Reference					
Teaching	1639.64	[−2697.30–5976.59]	0.459	−0.22	[−2.15-1.70]	0.819
**HOSPITAL BED CAPACITY**						
Small	Reference					
Medium	5096.48	[−1453.24–11646.2]	0.127	0.51	[−2.35–3.37]	0.727
Large	7446.41	[1527.81–13365.01]	0.014	0.6	[−1.57–2.77]	0.587
